# The effectiveness of the peer-delivered Thinking Healthy PLUS (THPP+) Program for maternal depression and child socioemotional development in Pakistan: study protocol for a randomized controlled trial

**DOI:** 10.1186/s13063-016-1530-y

**Published:** 2016-09-08

**Authors:** Elizabeth L. Turner, Siham Sikander, Omer Bangash, Ahmed Zaidi, Lisa Bates, John Gallis, Nima Ganga, Karen O’Donnell, Atif Rahman, Joanna Maselko

**Affiliations:** 1 Duke Global Health Institute, Duke University, Durham, USA; 2 Department of Biostatistics and Bioinformatics, Duke University, Durham, USA; 3 Human Development Research Foundation, Islamabad, Pakistan; 4 Department of Epidemiology, Columbia University School of Public Health, New York, USA; 5 Institute of Psychology, Health and Society, University of Liverpool, Liverpool, UK; 6 Department of Epidemiology, Gillings School of Public Health, University of North Carolina Chapel Hill, Chapel Hill, USA

**Keywords:** Thinking healthy program, Psychological treatment, Peer volunteers, Nonmental health professionals, Perinatal depression, Maternal depression, Task-shifting, Randomized trials, Low- and middle-income countries, Child development

## Abstract

**Background:**

The negative effects of perinatal depression on the mother and child start early and persist throughout the lifecourse (Lancet 369(9556):145–57, 2007; Am J Psychiatry 159(1):43-7, 2002; Arch Dis Child 77(2):99–101, 1997; J Pak Med Assoc 60(4):329; J Psychosoma Res 49(3):207–16, 2000; Clin Child Fam Psychol Rev 14(1):1–27, 2011). Given that 10–35 % of children worldwide are exposed to perinatal depression in their first year of life (Int Rev Psychiatry 8(1):37–54, 1996), mitigating this intergenerational risk is a global public health priority (Perspect Public Health 129(5):221–7, 2009; Trop Med Int Health 13(4):579–83, 2008; Br Med Bull 101(1):57–79, 2012). However, it is not clear whether intervention with depressed women can have long-term benefits for the mother and/or her child. We describe a study of the effectiveness of a peer-delivered depression intervention delivered through 36 postnatal months, the Thinking Healthy Program Peer-delivered PLUS (THPP+) for women and their children in rural Pakistan.

**Methods/design:**

The THPP+ study aims are: (1) to evaluate the effects of an extended 36-month perinatal depression intervention on maternal and index child outcomes using a cluster randomized controlled trial (c-RCT) and (2) to determine whether outcomes among index children of perinatally depressed women in the intervention arm converge with those of index children born to perinatally nondepressed women. The trial is designed to recruit 560 pregnant women who screened positive for perinatal depression (PHQ-9 score ≥10) from 40 village clusters, of which 20 receive the THPP+ intervention. An additional reference group consists of 560 perinatally nondepressed women from the same 40 clusters as the THPP+ trial. The women in the nondepressed group are not targeted to receive the THPP+ intervention; but, by recruiting pregnant women from both intervention and control clusters, we are able to evaluate any carryover effects of the THPP+ intervention on the women and their children. Perinatally depressed women in the THPP+ intervention arm receive bimonthly group-based sessions. Primary outcomes are 3-year maternal depression and 3-year child development indicators. Analyses are intention-to-treat and account for the clustered design.

**Discussion:**

This trial, together with the reference group, has the potential to further our understanding of the early developmental lifecourse of children of both perinatally depressed and perinatally nondepressed women in rural Pakistan and to determine whether intervening with women’s depression in the perinatal period can mitigate the negative effects of maternal depression on 36-month child development.

**Trial registration:**

THPP-P ClinicalTrials.gov Identifier: NCT02111915 (registered on 9 April 2014).

THPP+ ClinicalTrials.gov Identifier: NCT02658994 (registered on 21 January 2016).

Sponsor: Human Development Research Foundation (HDRF).

**Electronic supplementary material:**

The online version of this article (doi:10.1186/s13063-016-1530-y) contains supplementary material, which is available to authorized users.

## Background

Perinatal maternal depression, defined by at least one depressive episode during pregnancy and/or the first postnatal year, has been shown to have negative health effects for both the mother and the child. Negative effects on the mother include reductions in daily functioning as well as early mortality. Negative effects on the child, including illness and poor growth, start early and persist throughout the child’s life [1–6]. Given that 10–35 % of children worldwide are exposed to perinatal depression in their first year [7], mitigating this intergenerational risk is a global public health priority [8–10]. Pakistan has one of the highest rates of maternal depression globally, and one of the only studies examining potential long-term benefits of maternal depression interventions on child outcomes found no significant effects [11].

The Thinking Health Program (THP), a community health worker (CHW)-delivered intervention developed and evaluated in Pakistan, was shown to have beneficial effects on both perinatal maternal depression and short-term child outcomes including reductions in diarrheal episodes and increased vaccination rates [12]. In 2015, the THP was formally designated by the World Health Organization (WHO) as an evidence-based intervention that could be implemented in a variety of global settings using an established CHW healthcare delivery system [13]. Unfortunately, many CHW systems, such as Pakistan’s, are underfunded and stretched to capacity; and alternative delivery methods are required. In response to this need, the Thinking Health Program Peer-delivered (THPP) was developed by adapting the THP to be delivered primarily by peers who operate within the existing CHW system. An ongoing study, the THPP-Pakistan trial [14], seeks to evaluate THPP for 6 postnatal months.

Although effective in reducing maternal perinatal depression, our recent work failed to show that the 6-month CHW-led THP led to improved longer-term child outcomes [11]. At age 7 years, children of perinatally depressed mothers who received the intervention did not show better outcomes than children of control group mothers. To improve the longer-term outcomes of both perinatally depressed mothers and their children, we have developed the Thinking Health Program Peer-delivered PLUS (THPP+), an extension of the 6-month THPP intervention delivered at a lower intensity for an additional 30 postnatal months to the same women who have been receiving the THPP. The THPP+ is an extension and a continuation of the THPP intervention for mothers until the child is 3 years old.

The aim of this manuscript is to describe the protocol for the THPP+ study in Pakistan. The THPP+ study is a cluster randomized controlled trial (c-RCT), which compares outcomes among three groups of mother-child dyads: (1) those receiving the intervention, (2) those receiving Enhanced Usual Care (EUC) in the control clusters, and (3) a reference group of mother-child dyads in which the woman was not depressed in pregnancy and resides in the same intervention and control clusters where the trial is being implemented. Focusing on outcomes at 36 postnatal months, the goal of this c-RCT is to evaluate the cumulative effectiveness of the combined THPP and THPP+ interventions on mothers and their children. The goal of the embedded reference group of perinatally nondepressed women and their children is two-fold: (1) to evaluate whether the intervention is able to meaningfully reduce the gap in child outcomes that is traditionally observed when comparing children of depressed and nondepressed mothers; and (2) to determine whether there are any beneficial carryover effects of the intervention on this nondepressed group.

This manuscript complements and extends the THPP trial protocol [14]. To ensure that the current protocol is able to stand alone, we present the necessary key features of the THPP design and the ways in which the THPP+ trial builds on, and is different from, the ongoing THPP trial in Pakistan.

### Objectives and hypotheses

The primary objective of the study is to evaluate the impact of a 36-month perinatal peer-delivered community-based perinatal depression intervention on (1) maternal depression and (2) child development. Our primary hypothesis for the perinatally depressed mothers is that the intervention will result in lower prevalence of depression at 3 years postnatal. Our primary hypothesis for the children is that the perinatal depression intervention will lead to improved developmental outcomes (see “Measures and constructs” in Table 1) at 3 years of age. Additional child hypotheses address proposed mediators and moderators of the effects of the perinatal depression intervention on child outcomes.

**Table 1:** Primary outcome measures for women and children in the Thinking Health Program Peer-delivered PLUS (THPP+)

	Source of data	Postnatal months				
Outcomes	Measure	3	6	12	24	36
Mother: depression	Patient Health Questionnaire (PHQ-9)			✓	✓	✓
	WHO Disability Assessment Schedule (WHO-DAS)			✓	✓	✓
Child: socioemotional	Total Difficulties score from the Strengths and Difficulties Questionnaire (SDQ-TD)					✓
	Ages and Stages Questionnaire (ASQ)		✓	✓	✓	✓
Child: developmental milestones	Bayley Scales of Infant and Toddler Development III (BSITD-III)			✓	✓	✓
Child: physical	Length, weight (WHO weight-for-length *z*-scores)	✓	✓	✓	✓	✓
	Head circumference	✓	✓	✓	✓	✓
	Diarrhea/ARI		✓	✓	✓	✓

*ARI* acute respiratory infection, *WHO* World Health Organization

The second objective is to determine whether outcomes of perinatally depressed mothers and children in the intervention arm will converge to those in the reference group of perinatally nondepressed mothers and children as well as, secondarily, to determine whether there are any carryover effects of the intervention to benefit perinatally nondepressed mothers and children.

## Methods/design

### Trial settings

The study will be conducted in rural Pakistan in the rural Sub-District of Kallar Syedan, Rawalpindi, Pakistan.

### Design

The THPP+ trial is a stratified cluster randomized controlled trial (c-RCT) of 40 village clusters allocated in a 1:1 ratio to receive intervention or EUC within 11 strata defined by Union Councils (sub-district units), each with an even number of village clusters [15]. Cluster randomization is used to avoid contamination between women since the THPP intervention is delivered at the community level through CHWs and peer women in the community. Stratification is used to minimize imbalance in baseline covariates.

**Fig. 1:**
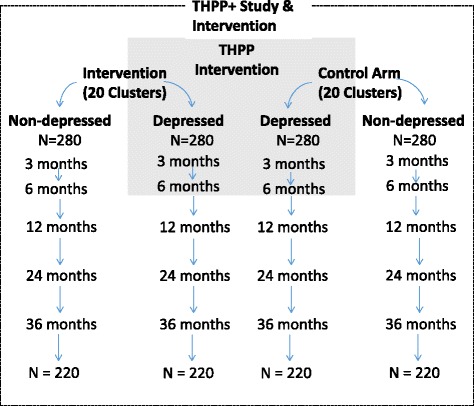
THPP+ study (both the trial of perinatally depressed mothers and the reference group of perinatally nondepressed mothers) in relation to the THPP intervention trial showing anticipated loss to follow-up. *N* = number of mother-child dyads. Unshaded area is unique to the THPP+ study

THPP+ is conducted in the same 40 village clusters as the THPP trial. The same study population of perinatally depressed women is invited to consent to participate in THPP+. An equal number of perinatally nondepressed women are also recruited from each village cluster. The latter forms the reference group that enables us to evaluate whether convergence of maternal and child outcomes occurs during the 3-year postnatal period. In summary, all depressed women enrolled in the THPP+ trial were enrolled in THPP, while all nondepressed women are only recruited to the THPP+ study. See Fig. 1 for details of the distinction.

In brief, the ongoing THPP trial focuses on the effects of the THPP intervention on maternal outcomes at 6 postnatal months, with a limited number of child outcomes measured. The THPP+ protocol is designed to recruit the same 560 pregnant women who screen positive for perinatal depression from the 40 village clusters described above for the THPP trial, of which 20 clusters receive the THPP intervention delivered by trained lay peer volunteers.

### Participants and procedures

**Fig. 2:**
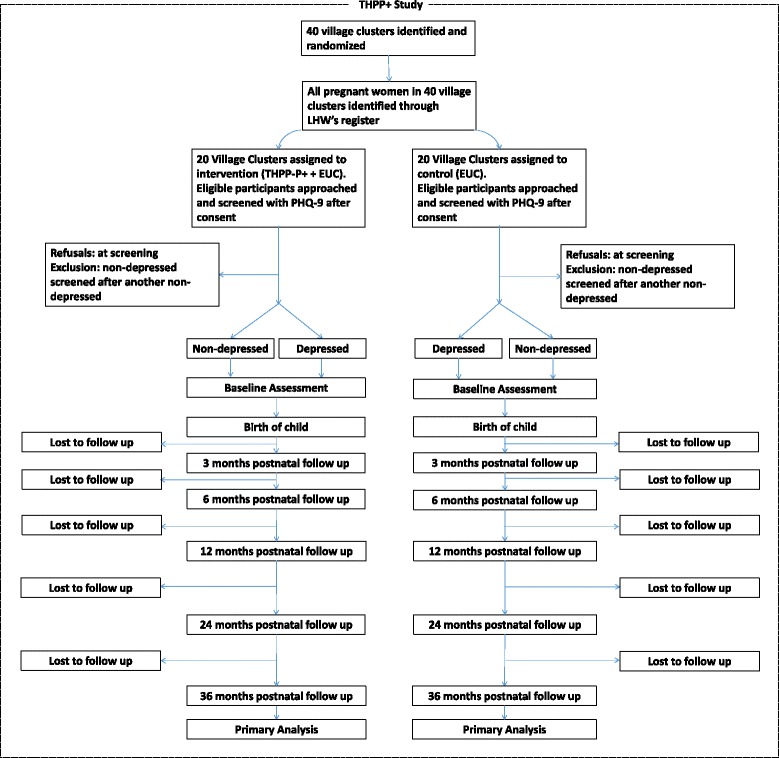
Flow chart of the THPP+ study

Figures 1 and 2 show recruitment and flow of both the perinatally depressed and perinatally nondepressed mother-child dyads through the study. After collecting prebirth baseline information, we assess each mother and her index child born during the study at 3, 6, 12, 24 and 36 postnatal months. The 3- and 6-month assessments will coincide with those of the THPP trial. The 12-, 24- and 36-month assessments are unique to THPP+ (further details in Additional file 1).

### Recruitment of study participants: inclusion and exclusion criteria

The current THPP+ study consists of perinatally depressed women and the index children who are participating in the THPP study and an additional sample of nonperinatally depressed mothers and of the index child of each mother. For THPP, pregnant women registered with the CHW (called Lady Health Workers) were approached. The study team has been engaged with the Lady Health Workers and the community in the past and enrollment rates have been consistently high. All eligible women in their third trimester of pregnancy were assessed for depression using the Patient Health Questionnaire (PHQ-9) and those scoring above the 10-point cutoff were invited to participate in the trial. For THPP, a random sample of approximately a third of women scoring less than 10 (i.e., screening negative on PHQ-9) are asked to serve as an additional reference group of equal size as the number of perinatally depressed women. In order to be eligible to participate, women need to be married, to reside in the study area, to understand one of the study languages (Urdu, Punjabi or Potohari), and to not require immediate medical attention. Following a live birth, the mother-infant dyads remain eligible to continue in the study unless the woman develops a psychotic or manic episode, or the dyad is broken through death, disability or relocation of the woman or child. Any participant who develops severe symptoms over the course of the study will be immediately referred for additional treatment.

### Informed consent

Women are informed about the study goals and study design in the third trimester of pregnancy by trained research staff. Those who agree to participate consent to be followed up for 3 years postnatally and to participate in an intervention if they screen positive for perinatal depression. This consent covers the THPP+ period. The additional THPP+ sessions are seamlessly added to the existing intervention content for depressed women in the intervention clusters.

### Randomization

The current THPP+ study is designed to maintain the randomization that was performed at the start of the THPP. According to the randomization procedure, 11 UC strata were selected with an even number of village clusters identified in each. Within each UC, village clusters were then randomized in a 1:1 ratio. In total, there are 20 intervention and 20 control arm clusters.

### Interventions

#### Thinking Healthy Program Peer-delivered (THPP)

The Thinking Healthy Program Peer-delivered (THPP) is an adaptation of the Lady Health Worker-delivered THP, that was adopted by the WHO mhGAP Series [13]. The protocol for the THPP trial has been published [14]. Similar in content to the THP, the peer-delivered version is simplified with additional strategies added for ease of implementation by peers. The intervention focuses on identifying and altering unhealthy behaviors with a focus on behavioral activation to facilitate change. It consists of both individual sessions with the peers as well as group sessions held at the “Health House,” a room in the home of the CHW dedicated for women’s group meetings. The THPP begins in the third trimester of pregnancy and finishes at the end of the sixth postnatal month.

#### Thinking Healthy Program Peer-delivered PLUS (THPP+)

As part of THPP+, the intervention continues from the beginning of the seventh postnatal month through the end of the 36th month, and consists of an additional 30 months of lower-intensity services unique to the THPP+ model. We use the term THPP+ to refer to the combined 6-month THPP intervention and the 30-month THPP+ intervention delivered consecutively through 36 months after the index child’s birth. The THPP+ includes group sessions to be held roughly every other month for a total of 18 sessions over the intervention duration. The content is a continuation of the previous THPP sessions with emphasis on self-care and on the baby’s health and development. In order to ensure continued participation, peers contact each woman a week prior to the group session and the groups are held in the community where the participants live and are easily accessible. Peers keep session logs which are overseen by the peer supervisors and can be used for calculation of “dose” during analyses.

In case a woman misses attending a session the peer follows up at the household to work out/negotiate with the family to ensure attendance at the next session (so that the “dose” is not missed).

Although the perinatally nondepressed women in the intervention arm do not receive the THPP+ intervention, by recruiting mothers from both intervention and control clusters, we are able to evaluate any effects of the THPP+ on the group that is not directly targeted.

### Enhanced Usual Care

Women in the control clusters who were depressed prenatally have been receiving Enhanced Usual Care (EUC). At the time of the screening (and with consent), women, their Lady Health Workers and personnel in their local primary health care facility were informed of the diagnosis; and women were given an information sheet about depression and how to access care. There are no new EUC protocols put in place postnatally as part of the THPP+.

### Additional training and supervision of peers

For the THPP, peers were trained in a 5-day classroom-based workshop, followed by a 2-month internship during which they practiced the content of the THPP on nontrial participants [14]. For THPP+, peers will receive an additional 2 days of classroom training after their last session (during the fifth postnatal month) of the THPP to cover the additional content. Competency is assessed by role plays. Peer counselors continue to receive monthly group supervision to maintain high motivation and to address any challenges in the field.

### Minimization of contamination

Risk of new contamination between the treatment and control arms is expected to be very low given the low intensity of the intervention and its placement after the end of the more intensive intervention that began prenatally and lasted through to 6 postnatal months. The cluster design makes it less likely that women will exchange information related to the intervention.

### Masking of treatment allocation

Although it is not possible to blind study participants from their treatment arm allocation, all project staff, including interviewers, are blind both to a woman’s original depression status and to the treatment arm of the village cluster in which she resides. Study participants are instructed to not discuss their depression status or intervention (or lack thereof) with the assessors. The data linking each village cluster with treatment allocation status is kept separate from the remaining outcome dataset until the time of the final analysis.

### Fidelity of the intervention

Fidelity of the intervention is assessed through documenting the number of women who attend the meetings in combination with documenting the content covered during the meetings and the duration of each component covered in the session.

### Data management

All data capture is performed electronically on tablets and uploaded daily to the main server. Quality checks for consistency, accuracy, missing data and other irregularities are conducted weekly. Any issues are shared with the research team and discussed during a weekly staff meeting to address source of any problems in the field. Data are backed up daily. Data are deidentified/anonymized before being shared with coinvestigators outside of the Human Development Research Foundation (HDRF). At all stages, data are password-protected with multiple layers of authorization.

### Outcome evaluation

The primary endpoint is designed to be at 3 years postnatally. The primary comparison tested is between perinatally depressed-intervention versus perinatally depressed-control women in order to evaluate the effectiveness of the THPP+ intervention on long-term outcomes in perinatally depressed mothers and their children born during the intervention period (i.e., “index child”). Secondary comparisons for mothers and their index children are (1) intervention perinatally depressed mothers versus control perinatally nondepressed mothers to assess convergence of outcomes in both mothers and children and (2) intervention perinatally nondepressed versus control perinatally nondepressed mothers to assess whether there are any carryover effects of the intervention that benefit perinatally nondepressed mothers and their index children. For the former, the statistical goal is to demonstrate equivalence of outcomes of control perinatally nondepressed and intervention perinatally depressed mothers and their children. For the latter, the goal is to test the null hypothesis of no difference between groups in the outcomes of interest. The mother and child outcome measures are detailed in Tables 1 and 2.

**Table 2:** Outcome assessments

Instrument	Description	Outcome	Contextual validity
PHQ-9	Nine-item questionnaire assessment of depressive symptoms assessed on a scale of 0 to 3	Prevalence of moderate–severe depression; mean total score	Validated in primary care [37]
WHO-DAS	12-item questionnaire for measuring functional impairment over the last 30 days. In addition, two items assess the number of days the person was unable to work in these 30 days	Total disability score; quality-adjusted life years; number of days out of work	Validated for international use [18]
SDQ-TD	The SDQ is a parent report of 25 child attributes divided into five subscales: emotional symptoms, conduct problems, hyperactivity, peer problems and prosocial behavior	Total Difficulties score: calculated based on four subscales (except prosocial behavior)	The SDQ has previously been translated into Urdu as well as at least 50 other languages and used in low- and middle-income countries [21–23]
ASQ	The ASQ is a widely used, simple set of 30 questions appropriate for 4–60 month-olds that assesses five domains of development	The total score from the five domains, plus the score from an additional domain on the child’s socioemotional development	The parent-report-based ASQ assessments have been shown to have good concurrent validity with professionally administered BSITD [24, 38], including internationally [39, 40]
BSITD-III	An individually administered assessment of the child’s achievement of developmental milestones across five areas: cognitive, language, motor, social-emotional and adaptive skills [27]	The total score from each domain	The standard scores are derived from the US norms; and, because there are no available Pakistani norms, the scores provide a metric with which to compare groups of children in this Pakistan setting relative to the study hypotheses

*ASQ* Ages and Stages Questionnaire Socio-Emotional scale, *BSITD-III* Bayley Scales of Infant and Toddler Development, Third Edition, *PHQ* Patient Health Questionnaire, *SDQ-TD* Strengths and Difficulties Questionnaire, *WHO-DAS* WHO Disability Assessment Schedule

### Mother outcome measure

#### Patient Health Questionnaire (PHQ-9)

The PHQ-9 is the main indicator of depression symptoms among the women in the study. The PHQ-9 inquires about frequency of depressive symptoms in the last 2 weeks. It has been validated and used extensively in the region [16, 17].

#### WHO Disability Assessment Schedule (WHO-DAS)

The WHO-DAS is a 12-item questionnaire assessing levels of function over the last 30 days. Combined with two items about one’s ability to work in the last 30 days, the WHO-DAS generates a total disability score, quality-adjusted life years and number of days the respondent is not able to work [18].

### Child outcome measures

#### Socioemotional development

Our main outcome measure is the Total Difficulties (TD) score derived from the Strengths and Difficulties Questionnaire (SDQ). The SDQ is a parent report of 25 child attributes divided into five subscales: emotional symptoms, conduct problems, hyperactivity, peer problems and prosocial behavior [19]. The TD score is calculated based on four subscales (except prosocial behavior) with a score range of 0–40 points [20]. The SDQ has previously been translated into Urdu as well as at least 50 other languages and used in low- and middle-income countries [21–23].

#### ASQ

Socioemotional developmental milestones, prior to and including 36 months, are assessed with the Ages and Stages Questionnaire Socio-Emotional scale (ASQ-SE) [24, 25]. The ASQ is a widely used, simple set of 25 questions where parents are asked to report age-appropriate milestones with the help of simple examiner-administered examples, such as whether, at 8 months, the child plays with a toy by banging it up or down on the floor or table [26].

#### Infant developmental milestone achievement

##### Bayley Scales of Infant Development

The Bayley Scales of Infant and Toddler Development, Third Edition (BSID-III) is an individually administered assessment of the child’s achievement of developmental milestones across five areas: cognitive, language, motor, social-emotional and adaptive skills [27]. The evaluations are conducted in the family’s home at infant ages 12, 24 and 36 months. Raw scores in each domain are summarized by chronological age-related scaled scores and composite scores for each domain. The standard scores are derived from the US norms; and, because there are no available Pakistani norms, the scores provide a metric with which to compare groups of children in this Pakistan setting relative to the study hypotheses. The evaluators were trained in administration of the BSID-III by the team clinical psychologist (O’Donnell, US-based) and by the local team, which includes a psychiatrist and a physician. Periodic quality assurance is assessed at least quarterly by dyadic testing (evaluator plus team psychologist) and by double scoring by the US-based psychologist.

#### Physical development

Physical development is assessed using weight-for-age and height-for-age. Weight-for-age is sensitive to weight change over a short time period but fails to distinguish tall, thin children from those who are short with adequate weight. Height-for-age is useful for identifying children with short stature, a group often vulnerable to longer-term adverse conditions. Based on WHO norms, a measure of 2 standard deviations (SD) below the mean of either weight or height is chosen to indicate poor growth. Head circumference is measured through 24 months. Physical health indicators are recent diarrheal episodes and acute respiratory infections.

### Power calculations

The primary power calculations for the THPP+ study are for the c-RCT comparisons of perinatally depressed women and their children in the control versus intervention arms at 36 postnatal months at the 5 % two-tailed significance level. As for the THPP trial [14] we assume 40 village clusters randomized in a 1:1 allocation ratio within 11 UCs, with 14 perinatally depressed women per village cluster, to yield a total sample size of 560 perinatally depressed women at baseline. In addition, for THPP+ we recruit 14 perinatally nondepressed women per village cluster for a total of 560 perinatally nondepressed women at baseline. We conservatively estimate that loss to follow-up (including infant mortality and maternal illness and death) of both perinatally depressed and perinatally nondepressed women at 36 months will be 20 % (anticipated loss to follow-up in the THP trial was 10 % at 6 months and most loss to follow-up is expected in the first 6 months of the study) [12]. Therefore, the total sample size available at 36 months is anticipated to be 480 perinatally depressed and 480 perinatally nondepressed women and their children. Using a standard formula [28, 29] for a cluster randomized design and assuming an intracluster correlation of 0.07 in the intervention arm and 0.05 in the control arm, the trial will have 90 % power at 36 months to detect a difference in perinatally depressed remission of 65 % in the perinatally depressed-intervention versus 45 % in the perinatally depressed-control for the anticipated total sample size of 480 perinatally depressed women at 36 months. For child outcomes, this sample size will yield power of more than 90 % to detect a difference between arms in mean TD score (range 0–40) of 3 points for children of perinatally depressed mothers using plausible estimates for intracluster correlations of 0.04–0.08 [12], and 5.2 for SD for the TD score among 3 year-olds [30].

Secondary comparisons mainly focus on child outcomes and are well-powered. For the secondary hypothesis of equivalence between children of perinatally depressed mothers in the intervention arm and perinatally nondepressed mothers in the control arm, we will conclude equivalence if the 95 % confidence interval (CI) for the difference between the mean score in the two groups lies between −2 and 2 units. We note that differences of 1.0–2.0 points are often observed between boys and girls [30, 31]. With 220 children in each group and conservatively assuming an overall significance level of 2.5 % (corresponding to the 95 % CI), an SD of 5.2 and an ICC of 0.04, and no difference between the groups, we will have 83 % power to conclude equivalence [28, 32]. For the secondary research question of the community benefit (i.e., carryover) of the intervention for perinatally nondepressed women and their children, we will have 80 % power to detect a 1.7 or greater impact of the intervention on mean TD score (groups: perinatally nondepressed-intervention versus perinatally nondepressed-control, Fig. 1) for the same assumptions of the primary comparison above.

### Analysis

Statistical analysis will be conducted according to the Consolidated Standards of Reporting Trials (CONSORT) guidelines. A flow chart will show participation of both perinatally depressed and perinatally nondepressed mothers and their children from recruitment in the third trimester through to 36 postnatal months (Fig. 2). Withdrawals and loss to follow-up will be shown at each follow-up (3, 6, 12, 24 and 36 postnatal months). Baseline characteristics of recruited mothers will be reported by study arm, and separately for perinatally depressed and perinatally nondepressed mothers. Continuous variables will be summarized by means and standard deviation (SD), or medians and the 25th and 75th percentile, if needed. Categorical variables will be summarized by counts and percentages.

The primary analyses are designed as intention-to-treat and will be conducted using the latest release of Stata software. Separate outcome analysis will be conducted for mothers and for children. In both cases, data from perinatally depressed and perinatally nondepressed participants will be analyzed jointly using generalized linear mixed-effects models so that all comparisons of interest can be estimated from the same model. The identity link will be used for continuous outcomes in order to estimate differences in mean outcomes. The log-link will be used for binary outcomes in order to estimate prevalence ratios, but if convergence is not achieved we will use the logit link from which prevalence ratios will be estimated. Random intercepts for cluster will be included to account for the clustered study design. For outcomes measured at multiple follow-up time points (e.g., for depression status in both perinatally depressed and perinatally nondepressed mothers, which will be evaluated at all five follow-up time points), random intercepts for person will be added to account for correlation of repeated measures on person. Similarly, in this case, random slopes for both cluster and participant will be considered to allow for heterogeneity by cluster and participant over time. All random error terms will be assumed independent and zero-mean normally distributed.

Primary analyses of outcomes measured at a single follow-up time point are designed to include the following fixed-factor variables: arm (intervention versus control), strata (11 Union Councils), baseline depression status (perinatally depressed versus perinatally nondepressed) and its interaction with arm. For outcomes measured at multiple follow-up time points, the interactions between study arm, follow-up time point and baseline depression status will be included to allow for different intervention effects at each follow-up time point. Estimates of the prespecified comparisons of interest will be derived from the fitted model. Conclusions about the equivalence of perinatally depressed-intervention and perinatally nondepressed-control will be based on whether the corresponding 95 % CI is contained within the equivalence margins (i.e., −2 to 2 for the primary child outcome of the TD score). Model assumptions will be assessed; in the case of non-normally distributed residuals, we will consider bootstrapping or transformations to obtain valid CIs.

Secondary analyses will include any baseline covariates for which there was chance baseline imbalance and for any additional baseline covariates that predict missing outcome data. Under the assumption that those covariates explain the missing data mechanism, we will obtain valid estimates of the intervention effects using the complete case data (i.e., without the need for imputation or an alternative method) [33]. If there are concerns or evidence that covariates cannot explain the nature of the missingness (i.e., if the data are missing not at random), we will perform a series of sensitivity analyses based on the pattern mixture approach [34].

#### Moderator and mediator analyses

In addition to our main outcomes, auxiliary analyses focus on potential moderators and mediators of any main associations. A-priori variables that might impact the degree to which the intervention affects depression symptoms include socioeconomic status, household composition, and the presence of interpersonal violence. These associations will be examined by including an interaction between the variable of interest and the intervention indicator in the primary outcome model. Potential mediators of interest include maternal responsiveness, the mother-child relationship and social support.

#### Compliance analysis

We plan to gather information on compliance with the intervention and evaluate whether there is any evidence of contamination between treatment arms.

### Trial management

Trial monitoring procedures are a continuation of procedures and infrastructure in place for the THPP. This includes oversight by two committees: the Trial Management Committee (TMC), which is charged with close monitoring of all aspects of the trial and its progress and the Trial Steering Committee (TSC), which will provide additional guidance on the overall trial protocols as well as oversee trial safety issues. The TMC is composed of the principal investigators and the site team (project director, data manager/trial manager, local outcome assessment trainer); it meets weekly. The TSC is composed of the principal investigators, study coinvestigators, the trial manager and the study statistician; the TSC will meet every 6 months.

### Ethical considerations

We protect the confidentiality of personal data principally through procedures to separate study data and participant identifiable data. Quantitative data gathered with the tablets for each participant at baseline retain personal identification items to minimize errors in transcribing identities, but these will be removed before transferring the data to Stata for analysis. We monitor the occurrence of a number of specific serious adverse events (SAEs) beyond the THPP trial (among the depressed cases); these include death of the participant or her child due to any cause, suicide attempt, hospital admission due to a psychiatric problem, and hospital admission of participant or infant due to a serious medical emergency. Their detection and appropriate response (involving an independent psychiatrist responding) will be reported to the local Ethics Committee. These SAEs are compiled by the data manager and a blinded summary report is shared with the principal investigators and the TSC.

## Discussion

This trial and the parallel reference group of perinatally nondepressed women have the potential to further our understanding of the early developmental lifecourse of children of both women who were, and were not, perinatally depressed and to evaluate whether intervening on mothers’ perinatal depression can mitigate the negative effects of maternal depression on child development at 36 months. By beginning our study in the third trimester of pregnancy and following the mother-child dyads with multiple assessments through 36 postnatal months we will be able to analyze the relationship between changes in maternal depressive symptoms and child outcomes. For example, we will be able to analyze the impact of early versus late remission; remission of symptoms followed by recurrence; and new onset of symptoms on child outcomes. With the 3 years of follow-up with multiple assessments, we will be able to undertake an analysis of potential time-varying mechanisms.

By also enrolling a group of women who were perinatally nondepressed we are additionally be able to address two substantive questions. The first is: How much of the risk due to maternal depression exposure can the intervention mitigate? We ultimately want to know whether the intervention can prevent the intergenerational transmission of negative mental health outcomes. The children of prenatally depressed mothers in both intervention and control arms of the THPP+ intervention study are at high risk for multiple adverse outcomes. We expect that, at the end of the study, the children in the intervention arm will be at lower risk. However, the full impact of the intervention can only be discerned if we know the level of risk remaining – that is, the difference between the reduced level of risk among children (of prenatally depressed mothers) in the intervention arm and the risk among children whose mothers were not depressed to begin with. If outcomes of these two groups are comparable, we can infer that the intervention may prevent the intergenerational transmission of risk. Unlike in high-income country settings, normative data for such a comparison does not exist in many low-resource areas, including Pakistan; hence, the enrollment of nondepressed women [35, 36]. The second substantive question is: Does the intervention have an impact on mothers and children living in the intervention clusters, even if the mother was not depressed prenatally? The community intervention was originally designed to improve outcomes among depressed women. However, we suspect that its design may lead to broader, population-wide effects.

By working in a rural setting in Pakistan and by combining the cohorts of perinatally depressed mothers in the c-RCT and nonperinatally depressed mothers, the THPP+ study offers a unique opportunity to understand, and to potentially help to mitigate, the effects of perinatal depression on both the mother and the child.

### Trial status

The THPP trial (and hence the THPP+ trial) began recruitment of participants in October 2014. Based on our previous work and pilot results with an approximately 25 % rate of perinatal depression, we expect to recruit the sample by the end of February 2016. The endpoint assessments of all the participants at 36 postnatal months will be completed by end 2018.

## Abbreviations

ASQ, Ages and Stages Questionnaire; BSID-III, Bayley Scales of Infant and Toddler Development (III); CHW community health worker; CI, confidence interval; c-RCT, cluster randomized controlled trial; EUC, Enhanced Usual Care; HDRF, Human Development Research Foundation; ICC, intracluster correlation; PHQ, Patient Health Questionnaire; SAE, serious adverse events; SD, standard deviation; SDQ, Strengths and Difficulties Questionnaire; TD, Total Difficulties (score of SDQ); THP, Thinking Healthy Program; THPP, Thinking Healthy Program Peer-delivered; THPP+, Thinking Healthy Program Peer-delivered PLUS; TMC, Trial Management Committee; TSC, Trial Steering Committee; UC, Union Council; WHO-DAS, WHO Disability Assessment Schedule

## Additional file

Additional file 1: SPIRIT study timeline. (DOC 46 kb)
