# Erratum to: The effectiveness of the peer delivered Thinking Healthy Plus (THPP+) Programme for maternal depression and child socio-emotional development in Pakistan: study protocol for A three-year cluster randomized controlled trial

**DOI:** 10.1186/s13063-016-1665-x

**Published:** 2017-02-16

**Authors:** Elizabeth L. Turner, Siham Sikander, Omer Bangash, Ahmed Zaidi, Lisa Bates, John Gallis, Nima Ganga, Karen O’Donnell, Atif Rahman, Joanna Maselko

**Affiliations:** 1 Duke Global Health Institute, Duke University, Durham, USA; 2 Department of Biostatistics and Bioinformatics, Duke University, Durham, USA; 3 Human Development Research Foundation, Islamabad, Pakistan; 4 Department of Epidemiology, Columbia University School of Public Health, New York, USA; 5 Institute of Psychology, Health and Society, University of Liverpool, Liverpool, UK; 6 Department of Epidemiology, Gillings School of Public Health, University of North Carolina Chapel Hill, Chapel Hill, USA

## Erratum

Unfortunately, the original version of this article [1] contained an error. An error was introduced to the original title of this article.

The title was incorrectly published as:

The effectiveness of the peer-delivered Thinking Healthy PLUS (THPP+) Program for maternal depression and child socioemotional development in Pakistan: study protocol for a randomized controlled trial

The correct title is below:

The effectiveness of the peer delivered Thinking Healthy Plus (THPP+) Programme for maternal depression and child socio-emotional development in Pakistan: study protocol for a three-year cluster randomized controlled trial.
