# Supplementary material for: The effectiveness of the peer-delivered Thinking Healthy PLUS (THPP+) Program for maternal depression and child socioemotional development in Pakistan: study protocol for a randomized controlled trial
Source: Trials. 2016 Sep 8;17(1):442. doi: 10.1186/s13063-016-1530-y (PMC5017048; doi:10.1186/s13063-016-1530-y)
Supplement: Additional file 1: — SPIRIT study timeline. (DOC 46 kb) [file 13063_2016_1530_MOESM1_ESM.doc]

SPIRIT Figure.

|  | **STUDY PERIOD** | | | | | | |
| --- | --- | --- | --- | --- | --- | --- | --- |
|  | **Allocation of clusters** | **Enrolment of participants (3^rd^ trimester)** | **Post-allocation** | | | | |
| **TIMEPOINT** |  | **0** | ***3 mos.*** | ***6 mos.*** | ***12 mos.*** | ***24 mos.*** | ***36 mos.*** |
| **ENROLMENT:** |  |  |  |  |  |  |  |
| **Eligibility screen** |  | X |  |  |  |  |  |
| **Informed consent** |  | X |  |  |  |  |  |
| **Allocation** | X |  |  |  |  |  |  |
| **INTERVENTIONS:** |  |  |  |  |  |  |  |
| ***THPP+*** |  |  |  |  |  |  |  |
| ***Enhanced Usual Care*** |  | X |  |  |  |  |  |
| **ASSESSMENTS:** |  |  |  |  |  |  |  |
| ***Baseline variables: SES, family composition*** |  | X |  |  |  |  |  |
| ***Mother related outcomes: PHQ-9, WHO-DAS*** |  | X |  |  | X | X | X |
| ***Child related outcomes: SDQ, ASQ, BSITD, physical health*** |  |  | X | X | X | X | X |
